# Organizational Health Literacy in Facilities for People with Disabilities: First Results of an Explorative Qualitative and Quantitative Study

**DOI:** 10.3390/ijerph17082886

**Published:** 2020-04-22

**Authors:** Katharina Rathmann, Theres Vockert, Lorena Denise Wetzel, Judith Lutz, Kevin Dadaczynski

**Affiliations:** 1 Department of Nursing and Health Sciences, Fulda University of Applied Sciences, 36037 Fulda, Germany; theres.vockert@pg.hs-fulda.de (T.V.); lorena-denise.wetzel@pg.hs-fulda.de (L.D.W.); judith.lutz@pg.hs-fulda.de (J.L.); kevin.dadaczynski@pg.hs-fulda.de (K.D.); 2 Centre for Applied Health Sciences, Leuphana University Lueneburg, 21335 Lueneburg, Germany

**Keywords:** organizational health literacy, people with disabilities, inclusion, health care organizations

## Abstract

To date, studies on individual and organizational health literacy (OHL) in facilities for people with disabilities are scarce. Thus, the aims of this study are (1) to adapt an existing instrument for measuring organizational health literacy (OHL), namely, the “Health literate health care organization scale” (HLHO-10), to the context of facilities for people with disabilities, (2) to quantitatively examine characteristics of OHL, and (3) to qualitatively assess the definition and role of OHL by interviewing managers and skilled staff. An online study in Germany with *N* = 130 managers and skilled staff in facilities for people with disabilities was conducted, using the adapted HLHO-10 questionnaire. Univariate analyses were applied. Qualitative content analysis was used to investigate interview data from *N* = 8 managers and skilled staff from *N* = 8 facilities for people with disabilities in Hesse, Germany. Quantitative results revealed that respondents reported a below-average level in HLHO-10, with the lowest level found in the attribute of participative development of health information. The qualitative findings showed a clear need for improved navigation to and in facilities. The quantitative and qualitative findings are mainly consistent. Future research and measures should focus on facilities for people with disabilities in order to strengthen the development of and access to target-group-specific health information, as well as to establish a health-literate working and living environment.

## 1. Introduction

In early 2018, the National Action Plan on Health Literacy (NAP HL, [1]) was published in Germany, stressing the importance of individual skills and abilities in searching, understanding, evaluating, and applying health-relevant information [1]. There are numerous definitions of health literacy (HL) [2,3]. Common to all attempts at defining the term HL is the assumption that HL is indispensable to maintaining and promoting one’s own health, to coping with and overcoming diseases, and to “navigating” through the health system [2]. Limited HL is negatively associated with health, health behavior, treatment, and care services in the event of disease [3]. Further, it has been found that limited HL is related to more frequent hospital admissions and causes 3–5% of the costs in the health care system [4,5].

Previous surveys with representative samples in Germany have shown that 54% of the population in Germany reported limited HL and thus showed difficulties finding their way around the health care system and understanding relevant health-related information [6]. Findings of the HLS-EU survey showed that a total of 47.6% of the European population surveyed reported a limited HL. For instance, people in Bulgaria (62.1%), Spain (58.3%), Austria (56.4%), and Greece (44.8%) revealed a limited HL [6].

In Germany, a new field of research has emerged that investigates the HL of vulnerable population groups, including people with low socio-economic status, elderly people, and people with disabilities [5,7,8]. In particular, people with special needs and disabilities (including mental, physical, or psychological disabilities) are disadvantaged in many respects, such as education, employment, and health care [9,10,11]. Due to their (health) impairments, they are more likely to have an increased risk of concomitant and secondary diseases, while having a potentially higher need for long-term care beginning at a younger age compared to population groups without disabilities [10,12]. Previous studies also revealed that people with disabilities more frequently encounter barriers in access to and use of health care services, as well as use medical insurance benefits more frequently [10,13]. For instance, an explorative study showed that people with disabilities reported higher rates of limited HL compared to people without disabilities [8]. 

### 1.1. Organizational HL

Although the concept of individual HL is multidimensional [14,15], prior research has mainly focused on the concept of functional HL by Nutbeam and the concept of skills by Sørensen [16,17,18,19]. According to Nutbeam, functional HL consists of basic reading, literacy, and writing skills, and knowledge of health systems and conditions [14]. Sørensen et al. [20] defines HL as the “knowledge, motivation and competency to access, understand, appraise and apply health information in order to make judgments and take decisions in everyday life concerning healthcare, disease prevention and health promotion to maintain or improve quality of life during the life course”. Recent studies also reflected on the importance of enabling people to critically evaluate and question the quality of health information they find—also in relevant facilities, such as hospitals and health care organizations. The German NAP HL stressed a wider understanding of HL and now calls for health systems to become more user-friendly and health-literate at all levels, addressing the patient- or client-level, staff-level, and organizational level. In particular, health care organizations and all stakeholders should, thus, be involved in the promotion of HL [3]. 

In general, organizational HL (OHL) in health care (i.e., hospitals, health care organizations, and facilities for people with disabilities) includes all efforts to address individual HL of patients or clients and staff. OHL also refers to organizational conditions that help stakeholders to find their way in the health system and support those people with limited individual HL [21]. A wide definition of OHL by Brach et al. [22] comprises ten features of OHL: (1) A leadership that integrates HL as a substantial concept to its operations and all business activities; (2) HL is integrated into the planning, patient safety, evaluation, and quality management; (3) The staff is prepared to be health literate and supervises the progress; (4) Populations are involved in the development, implementation, and evaluation of health information and services; (5) The needs of persons with different ranges of HL skills are met while stigmatization is avoided; (6) HL is applied in the communication and understanding is confirmed at all points of contact; (7) Easy access to health information, services, and navigation assistance is provided; (8) Print, audio-visual, and social media contents, which are designed and distributed, are easy to understand and act on; (9) HL is also addressed in high-risk situations; and (10) Communicates clearly the costs for services.

The main aim of OHL is to establish the promotion of HL in all structures and processes within organizations [18,23]. Improving OHL is likely to enable health care organizations to be adapted to the needs of patients [17], to strengthen patient satisfaction [24], to improve the quality of care [25], and to provide equal access to health care tailored to the capacities and competences of the general population [26]. By doing so, not only can certain groups of people (e.g., those with limited HL) benefit from OHL measures, but all stakeholders in a particular setting [27]. 

### 1.2. Measurement of OHL, Interventions and Guides

Various (survey) instruments have been developed in order to assess attributes and degree of OHL, as well as to detect requirements for action in relation to OHL [4,28,29]: the HLHO-10 [28], the Health Literacy Universal Precautions Toolkit [29,30], and the V-HLO [31]. These tools examined various areas and attributes of OHL (e.g., HL awareness, verbal and written communication, or target-group self-management and empowerment) in order to assess the quality and potential of OHL more deeply [29]. Based on the 10 attributes of health-literate organizations [22], Kowalski et al. [28] developed in 2015 a valid and reliable measurement tool in the German language, including 10 items to self-assess the level of OHL, which were also validated in the English language. The so-called HLHO-10 can be used in two ways: (1) it can be applied in research to assess the level of OHL in hospitals or other health care organizations, and (2) it can be used as a self-assessment tool to identify areas with potential for improvement, as well as to develop measures to improve OHL. By using this instrument, progress in promoting OHL can be measured by a regular assessment.

Types of interventions have proved to be relevant for the improvement of OHL: institutional and partnership strategies, as well as individual actions [32]. Actions at an institutional level aim to create a favorable setting for the promotion of OHL by, e.g., defining and strengthening supportive norms, providing resources, and defining HL-promoting standards. Organizations with structurally anchored activities are those that have mandatory strategies for improving individual and organizational HL, while they prioritize OHL at all levels of the organization, especially at the management level. Interventions at the institutional level put the content of organizational measures into practice, indicating, for instance, facilitation of contact between patients/clients and (health care) organizations (e.g., by training staff and identifying particular weaknesses with regard to the HL of the target group and organization). Further, those measures to improve OHL can be located either at the individual level or at the organization-/system-level (e.g., [4]). At the individual level, this implies training and strengthening the personal competence of clients (e.g., by providing learning opportunities such as counselling, coaching, and training on health and HL). 

Guides of health-literate organizations differ greatly in their scope and context of application [33]. Particularly in English-speaking countries, there are numerous guides available to strengthen OHL [23,25,29,32]. It seems that guides on organizational health promotion are mainly located in health care organizations (i.e., hospitals) [18,21,27]. In contrast to studies on OHL in hospitals [18,21,27], there is rare evidence on OHL of organizations other than hospitals or health care organizations (such as youth work [16] or maternal and child health [34]).

### 1.3. State of the Art on OHL

Even though studies showed that measurement instruments such as the Health Literacy Universal Precautions Toolkit, V-HLO, and HLHO-10 have the potential to be applied to areas other than health care [16,22,27], the approach of health-literate organization (HLO) has not been transferred to and piloted in facilities for people with disabilities in Germany. To our knowledge, there is only one well-known large-scale research project in Germany that focuses on the promotion of OHL in care facilities and facilities for people with disabilities [35], which is conducted by a German health insurance company. The project on ‘Quality-oriented prevention and health promotion in organizations of integration assistance and care’ (QualiPEP) aims at developing, piloting, and implementing a quality framework for prevention measures and health promotion in inpatient, semi-inpatient and outpatient care, as well as in facilities for people with disabilities [35]. In this context, the promotion of HL is also oriented to people with disabilities, including both individual and organizational HL [36].

The majority of research on the implementation of OHL has focused on organizational measures promoting OHL, but insufficient consideration is given to organizational and partnership interventions [17,23,37]. Most interventions for the promotion of OHL are informally conducted in organizations and often emphasize the communication climate within (health care) organizations [18,32]. Regarding people with disabilities, the issue of health communication is particularly important. Improved communication, for instance, between clients and skilled staff, as well as better self-management, could reduce time, costs, and human resources for organizations [3,4,19,38,39].

Further, skilled staff in the organizations play a decisive role in the implementation of interventions on the promotion of OHL, as they are available as a trustworthy source of information for patients and clients [18,23,27,32,33,37]. In particular, interventions are recommended that make it easier, for example, for clients as well as other stakeholders, to access and find their way to and through the organization. Previous studies unanimously emphasized formal and, above all, organizational or organizationally anchored measures focusing on the living and working settings of staff members, while clients are particularly effective in establishing and strengthening OHL [5,17,23,25,40]. Those interventions include, for example, formal or organizational measures integrating OHL in all planning processes (e.g., new departments in the organization, organizational events), the provision of financial and human resources [33], and interventions tailored to the organization, such as the introduction of a structured feedback culture or a barrier-free guidance system [18]. It is also important to analyze the interfaces between the organization and other health care providers in order to identify further requirements, as well as to optimize, for example, transition of care. 

Prior research findings showed that there are a number of conditions for the successful implementation of HLO [23,25,33,37]. A systematic review by Farmanova et al. [33] revealed 13 different obstacles found in three central areas of organizations: (1) organizational culture and leadership, (2) design and implementation, and (3) human resources. These obstacles include, for example, a lack of awareness of the relevance of HL, an ambiguity in the roles and functions of staff with regard to HL, a lack of training in HL, a lack of commitment to HL, and a lack of support from leadership or management [33]. For instance, particular obstacles are limited time or human resources, as well as financial capacities [23,25,33]. In addition, the lack of guides or formal protocols to support practices that promote HL can hamper the process towards becoming a HLO [22,37]. In sum, previous studies highlighted that little is known about (a) characteristics of OHL in facilities for people with disabilities [10,35]. Further, there are—to our knowledge—no qualitative studies on (b) the definition and role of OHL in settings for people with disabilities in Germany.

### 1.4. Research Questions

The aims of this pilot study are to adapt and pilot the validated instrument of HLHO-10 to facilities for people with disabilities by (1) assessing the degree in 10 attributes of HLHO by an online survey (quantitative study) and (2) to examine the definition and role of OHL in facilities for people with disabilities (qualitative study).

The following research questions are addressed in this study:

1. How can the HLHO-10-instrument be appropriately adapted for its use in facilities for people with disabilities?
2. What is the degree of each of the 10 attributes of HLHO in facilities for people with disabilities? (quantitative study)
3. What is the definition and role of OHL in facilities for people with disabilities? (qualitative study)

## 2. Data and Methods

Due to the explorative nature of examining OHL in facilities for people with disabilities, the methodological approach of this study is based on a mixed-method approach according to Greene et al. [41]. The study followed a parallel design, i.e., a quantitative and a qualitative study was carried out simultaneously, while both studies were assigned the same priority. By combining qualitative and quantitative elements, it was possible to gain a detailed view of OHL in facilities for people with disabilities. The quantitative study provided insights into different dimensions of OHL in facilities for people with disabilities in Germany, while the qualitative study emphasized the definition and role of OHL from the perspective of skilled staff and managers. ‘Additional coverage’ [41] generates in-depth and additional knowledge about the findings of both parts of the study [42].

### 2.1. The Quantitative Study

#### 2.1.1. Database 

The quantitative study comprised a Germany-wide online survey on OHL in facilities for people with disabilities from mid-June to the end of July 2019. For this purpose, *N* = 2720 facilities for people with disabilities were contacted by e-mail throughout Germany. Facilities comprised inpatient, semi-inpatient, and outpatient residential facilities for people with disabilities (*N* = 1733), as well as sheltered workshops for people with disabilities (*N* = 987). Sheltered workshops for people with disabilities are workplaces were people with disabilities can be employed who are not able or have not yet found employment in the primary labor market because of the type or the extent of disability. People with disabilities can receive, in addition to the appropriate vocational training, a payment of a salary commensurate with the work performed. Further, employment in workshops makes it possible to maintain, develop, increase, or regain the ability to work or to be employed. Also, it serves for further development of the personality, as well as the promotion of the transition to the primary labor market. The online questionnaire took 28 to 35 min to complete. 

#### 2.1.2. Description of Instruments and Variables

The online questionnaire consisted of three parts. First, it contained information on features of facilities for people with disabilities, as did the qualitative interview study. In particular, data were gathered on the size of the organization (number of skilled staff, number of clients), the type of organization, financing (i.e., state, non-state, church, private) and profit orientation (yes vs. no). In this study, ‘clients’ were defined as all residents with a disability in facilities or employees with a disability in sheltered workshops. In order to differentiate between people with disabilities and those without, we used the term ‘skilled staff’ for respondents with a leadership position, managers, and skilled employees, as well as those employed in the housekeeping sector. Thus, the terms ‘skilled staff’ or ‘employees’ referred to those people without disabilities working in the investigated facilities.

The main purpose, represented in the second part of the questionnaire, was to assess the attributes of OHL, which were surveyed using the HLHO-10 questionnaire [28]. This instrument, which addresses the ten attributes of HLO [22], was developed by Kowalski et al. [28] in 2015 and has been tested as a valid and reliable measure in the German language including 10-items to self-assess the level of OHL. The following items on features of OHL are included: questions on the extent to which the organization devotes itself to HL in its mission statement, quality management, or HR planning; the application and development of (individualized) health information; the navigation and provision of various media in the organization; communication standards, particularly in critical situations; and the support of clients in health-relevant topics, as well as the training of skilled staff in HL. The seven-point response categories of each HLHO-10-item ranged from ‘very little’ (=1) to ‘very much’ (=7) in line with the original instrument. 

The third part of the questionnaire, which is not part of the analyses, assessed the implementation status of corporate health management in facilities. 

In relation to research question 1, the HLHO-10 instrument had to be adapted to the wording of facilities for people with disabilities. Only slight modifications to the HLHO-10 questionnaire were made after a pre-test involving two members with leading positions in two independent facilities for people with disabilities. One person was a board member of a sheltered workshop for people with disabilities; the other person was a skilled staff member with a leading position in a living residence for people with disabilities. No changes were made to the content of the questionnaire. Therefore, the adaption of the instrument HLHO-10 related mainly to the wording of the items, such as using the term ‘clients’ instead of ‘patients’.

#### 2.1.3. Study Sample

Of the *N* = 2720 facilities for people with disabilities, a total of *n* = 130 respondents from facilities took part in the online survey after two e-mail reminders. The response rate was 4.78%. As shown in Table 1, the sample is made up of 69.2% (*n* = 90) facilities for people with disabilities that are non-state or church organizations and 18.5% (*n* = 24) that are privately run and thus profit-oriented. 

Around 12% (*n* = 16) of the facilities for people with disabilities are publicly or municipally run. Almost 60% of the facilities have fewer than 50 skilled staff (*n* = 77), while just under 41% (*n* = 53) have more than 250 skilled staff. More than a quarter (26.2%, *n* = 34) are sheltered workshops. The largest share with 73.8% (*n* = 96) provides housing for people with disabilities. Of these, 66.2% (*n* = 86) are inpatient, 3.8% (*n* = 5) semi-inpatient, and 3.8% (*n* = 5) outpatient facilities (Table 1).

#### 2.1.4. Data Analysis 

Univariate analysis was conducted using all cases of *N* = 130 participants of the online survey. In more detail, frequency tables and mean values (with corresponding standard deviations) were extracted for single items of HLHO-10 and the overall index of HLHO in order to present the degree of the HLHO attributes. Analyses were conducted using the SPSS statistics (version 25) software.

### 2.2. The Qualitative Study

#### 2.2.1. Database 

The qualitative study was carried out in June 2019 in facilities for people with disabilities in the German federal state of Hesse. Theoretical sampling was used to select the facilities. The selection was based on facilities that showed the greatest possible heterogeneity (i.e., outpatient, inpatient, sheltered workshops, and facilities offering both housing and sheltered workshops for people with disabilities). First, an invitation letter was sent by e-mail, followed by phone calls by the project leader and team. Since the aim of the study was to examine the definition and role of OHL in facilities for people with disabilities in detail, we conducted explorative interviews. It was not the aim to systematize fields of action through systematic expert interviews or even to include subjective interpretations of the interviewees through theory-generating expert interviews [43]. The interviews were conducted with skilled staff in a leading function (i.e., organizational management, team management, etc., as well as executive leadership). Since the questions of HLHO-10 relate to the mission statement of the organization, employees without management or leadership functions were not interviewed, as they are likely to have limited or no precise knowledge of the development process or of the mission statement itself. Interviews lasted between 50 and 80 min. The qualitative part of the study was based on the international COREQ criteria (Consolidated criteria for reporting qualitative research) [44] and followed the standards of qualitative research [45]. The interviews were coded independently by two project staff members to increase the plausibility of the analysis. In case of inconsistency, they were discussed and debated by the entire project team. Prior to the start of the study, all participants were informed about the contents and objectives of the project and how to handle the collected data.

#### 2.2.2. Interview Guide

The interview guide was based on the items of HLHO-10 [28]. In the course of the interviews, the experiences and subjective perspectives of the managers and skilled staff on OHL were recorded. The focus was on their understanding and role of an HLO and the health-promoting structures in their facility. Possible obstacles to the design of HLO were also part of the interview guide. Since there are large differences among facilities for people with disabilities, the interview was preceded by a short questionnaire on socio-demographic features of the organization (e.g., the organization’s financing, human resource structure, such as number and type of skilled staff, and number of clients). This short questionnaire was also part of the quantitative study. 

#### 2.2.3. Study Sample

Of the 15 facilities for people with disabilities contacted via e-mail, *N* = 8 interviews were conducted with skilled staff in eight facilities for people with disabilities (outpatient housing *n* = 2; combination of housing and sheltered workshops for people with disabilities *n* = 2; sheltered workshops for people with disabilities *n* = 3; assisted living *n* = 1; see Table 1). Each type of facility should be represented at least twice. For this purpose, the interviews were conducted face to face with one skilled staff member in *n* = 6 of the facilities and *n* = 2 members in two facilities (managers *n* = 7; skilled staff *n* = 2; health management coordinator *n* = 1; executive leader *n* = 1) by two members of the project team. After informed consent from the interview partners, interviews were audiotaped. 

#### 2.2.4. Analysis 

**Figure 1:**
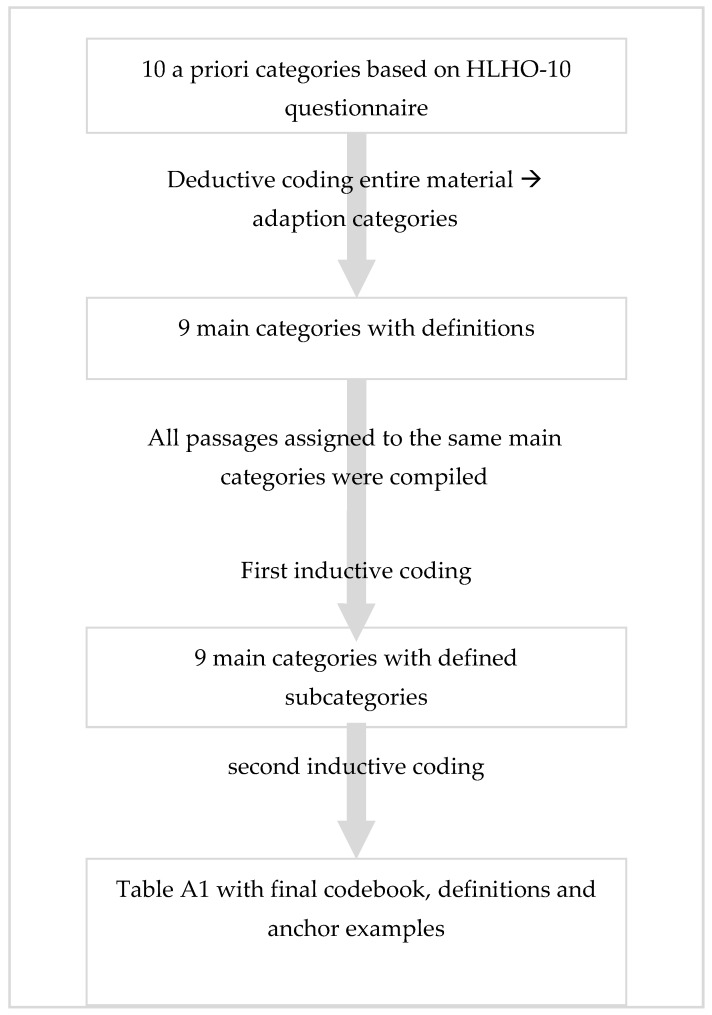
Coding procedure (separate display).

The interviews were transcribed by trained project members with the transcription program f4, following the content-semantic rules [46], and were double checked by project members. The qualitative content analysis was carried out following Kuckartz [42,46] using MAXQDA software (version 2018). All interviews were included in the analyses. Both a deductive and an inductive procedure were used for the two-stage coding process, which follows the procedure of content analysis (see Figure 1). The codebook was developed using so-called a priori categories based on the HLHO-10 questionnaire [28] for the first screening of the interview material (for each item of the HLHO-10, one a priori category was developed). 

In this deductive stage, these categories were used as a kind of search grid to test their fit to the empirical material and were then adapted. This procedure included several rounds of coding, as individual text passages could be assigned to several different codes. A unit could consist of several sentences as only meaningful units of text were given a code (i.e., the unit should be understandable outside of its context). Subsequently, all text passages assigned to the same category were compiled in preparation for the second stage of the coding process. 

**Figure 2:**
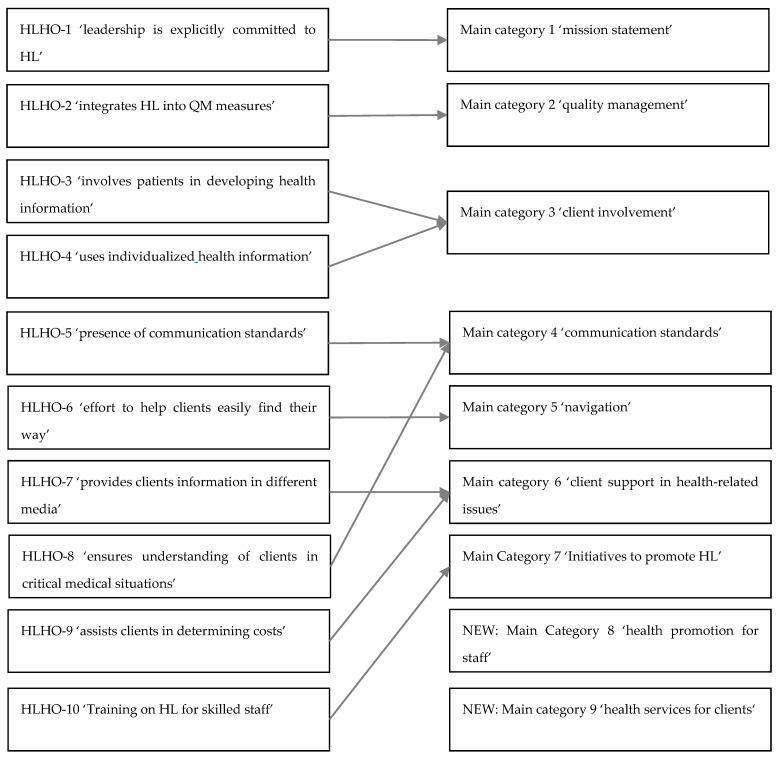
Changes from ‘a priori categories’ (HLHO-10 items) to main categories (quality content analysis).

The initial coding of the interviews tested the applicability of these categories to the empirical material. Even if, according to the literature, 10–25% of the empirical material is sufficient for determining thematic categories for the initial coding process [46], all data material was included in the coding and interpretation process. This is helpful to avoid losing information due to different types of organizations being studied. The a priori categories from HLHO-10 were found to be only partially suitable for analyzing the interviews as the aim of qualitative content analysis is to develop the main themes of the interviewees in relation to the questions asked. For example, the analysis of interview material showed that some HLHO-10 categories were considered by interviewees as more important than other properties of OHL. After deductive coding, nine categories from the interview material were created and the final codebook differed from the original HLHO-10 a priori categories. Differences between a priori categories and the final codebook are presented in Figure 2. The adapted categories (nine main categories) were used for a first coding of the data material. For all text passages that were assigned to one main category, a list of relevant topics related to this main category was generated. These relevant topics of one main category where used in an inductive procedure to create the subcategories for each of the main categories. This list of subcategories was compiled in exchange with the members of the project team and recorded in more general subcategories. Subsequently, definitions for these subcategories and illustrative anchor examples were formulated using quotations from the material. Table A1 (online) shows the codebook with definitions of the categories and anchor examples. These statements served as empirical data for analysis. 

The coding process of the interview material clarified that some codes were similar in terms of content and topic, while others were not. This procedure is based on guidelines for qualitative content analysis, in which main categories are more abstract and reflect thematic proximity [46]. The analysis of interviewees’ understanding of OHL was carried out separately without reference to the codebook. This means that all text passages from the entire qualitative interview material were compiled and then analyzed to generate topics allowing a better understanding of OHL.

## 3. Results

### 3.1. Results of the Quantitative Study 

**Figure 3:**
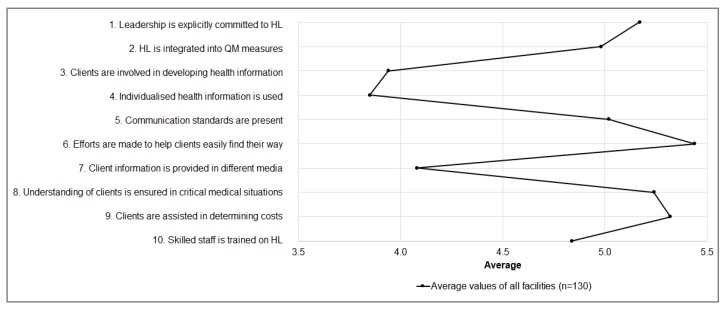
Mean values of the HLHO-10-items (*N* = 130). Note: HL = health literacy; QM = quality management.

Descriptive results were based on the adapted version of the HLHO-10 questionnaire to the context of facilities for people with disabilities. Figure 3 and Table 2 show the single items of the HLHO-10-questionnaire that were adapted to the wording of facilities for people with disabilities. Mean values of the HLHO-10-items are presented: on a scale of 1 (low level of OHL) to 7 (high level of OHL) the mean values for all single items range between 3.8 and 5.4. 

The average mean value of all items is 4.8 (standard deviation, SD = 1.1) (Table 2). “Health information developed with the involvement of clients (HLHO-3)” and the “use of individualized health information (HLHO-4)” scored the lowest. In contrast, highest values are found for “efforts to assist patients in finding their way around organization services” (M = 5.4, SD = 1.5) and the “assistance they receive from skilled staff in determining treatment costs” (M = 5.3, SD = 1.8). 

### 3.2. Results of the Qualitative Study

The results of the qualitative content analysis are presented below. With regard to the categories adapted from the HLHO-10 instrument, definitions and anchor examples of each main category and subcategory identified in the qualitative content analysis are presented in Table A1 (Appendix A). First, interviewees were asked about their general understanding of OHL in their facility for people with disabilities. Subsequently, results of each main category of OHL are presented. 

#### 3.2.1. Understanding the OHL of Facilities for People with Disabilities

Overall, interview partners reported a diverse and sometimes very complex understanding of OHL (Appendix A, Table A1). On the one hand, interviewees showed a basic understanding of OHL in the sense of prevention by promoting health-related behavior: ‘*Set up a cafeteria so that healthy eating is possible*’ (Facility for people with disabilities 1, hereafter FPD). To note: Quotes from the interviews were edited to improve readability. Other interviewees understood OHL as a matter of ‘training skilled staff’. Interviewees in a third type of FPD reported a more complex understanding of OHL. Interview partners in those facilities described OHL as a task of the facility itself to promote HL of clients and skilled staff. In addition to the promotion of health among clients, it was important for some interviewees that their facility provides resources (e.g., through doctors or therapists) to support clients and thus ‘*promote the mental and health development of our clients’* (FPD 2). It was also reported that it is important that clients know where they can get health relevant information in order to act independently. They can *‘get help when they realize that help is needed, when health situations from simple flu to serious diseases (...) affect them, when they can help themselves by consulting a doctor, when they get advice, when they (...) find ways and know where they can get help.’* (FPD 6). Further, skilled staff received training in health concepts and discounted membership in sports clubs or gyms in some FPD.

#### 3.2.2. Main Category 1: Mission Statement 

Interview partners reported—very heterogeneous and to varying degrees— that OHL is only partly reflected in some of the mission statements of the FPD. Four different forms of presenting OHL in the mission statement were identified. First of all, there were facilities that established HL in the mission statement (Table A1: 1.1). There are also facilities in which OHL is not an obligatory element, has relatively little importance, or has been integrated into the mission statement under a different name (1.2), as well as FPD that have not anchored HL in their mission statement (1.3). As a fourth type, there were FPD that implemented or offered OHL measures, although they did not conceptually define them as OHL. These facilities are considered to have implicitly anchored HL in their mission statement (1.4): *‘No, there are offerings that have simply grown over time out of (...) interests that some had or demands from others, but there is still no holistic concept.’* (FPD 3, person A). Among these cases are facilities with an anthroposophical orientation, that are institutions which have a very holistic view of humanity and aim at the gradual development of the human being towards free self-determination. In addition to the promotion of self-determination, a healthy development is also of central importance for those FPD. Here an understanding of HL is embedded in the holistic perception of humans and accordingly it is implicitly integrated in the mission statement. 

#### 3.2.3. Main Category 2: Quality Management 

In the area of quality management (QM), OHL is taken into account less frequently by FPD. Most facilities have a QM system in place. However, QM plays a more important role in sheltered workshops, clearly related to occupational safety and measures to improve and monitor working conditions and work schedules (2.1). Interviewees in sheltered workshops, for example, mentioned noise protection, ergonomics, or working time regulations as QM issues. For interviewees in other facilities reported to implement QM measures, OHL was not part of the QM. In critical medical situations, such as injuries to clients at the workplace, QM was of great relevance in all facilities. Almost all interview partners reported to have established routines in handling emergencies and complying with legal regulations. This is ensured by regular training courses, first aid courses, in-house emergency call systems, fixed procedures, or personal disease-specific emergency plans. Less frequently reported elements of OHL in QM were issues of social cooperation, potential conflicts in the workplace or in the care of clients, and related to medical care (2.2). The provision of medical care was clearly regulated by interprofessional cooperation and communication, as well as medication plans in some FPD. In those FPD professionals, skilled staff and some clients received proactive violence prevention or de-escalation training, or colleagues were trained for cases of traumatic events in the workplace. 

#### 3.2.4. Main Category 3: Client Involvement in Developing Health Information

Regarding client involvement in developing health information, interviewees reported provision of little to no information materials on health-relevant topics to their clients (3.2). Only one facility reported that it provides health information. Material on health information was usually developed by skilled staff in FPD, but not with the participatory involvement of clients. When the members of the facilities were asked about the participatory development of health information, their answers were mostly related to the development of health or leisure services, with focus on particular activities. In addition, skilled staff used their experiences to determine clients’ needs. Lastly, the implementation of health or leisure services strongly depended on the commitment of skilled staff (see Table A1). 

#### 3.2.5. Main Category 4: Communication Standards 

Communication between skilled staff and clients in FPD was clearly related to the individual situation and specific client (4.1). From the interviewees’ point of view, communication takes place among equals, is adapted to the individual recipient, and varies depending on each case. An obligatory communication standard at the organizational level was not reported by the participating interviewees. Further, ensuring client understanding is of central importance. The clients themselves, especially the level of disability and personal resources, are at the center of the communicative situation. *‘I would argue that there is no easy language that fits every client. (...) Actually, you’d need a language level for every single person you deal with.’* (FPD 2).

However, there are certain basic skills that are necessary for successful communication (4.3). This involves using certain communication techniques (including easy language and aids to comprehension such as pictograms). Easy language is a specially regulated simple language in German for people with especially intellectual disabilities. A set of rules is published by the German association “Netzwerk Leichte Sprache” [47]. These basic tools need to be carefully selected and adapted to the clients and the situation. For example, a critical medical situation can influence communication (4.2). In critical situations, the focus is always on the client’s understanding of the process and the situation. If a situation requires immediate action, the well-being and protection of both the client and the skilled staff, are at the center of the process and influence communication accordingly.

Interviewees revealed that communication is undertaken and effort is made to ensure clients’ understanding in all situations. From interviewees’ point of view, the basis for successful communication is to individually adapt communication to each individual client and not, as might be assumed, following existing communication standards. 

#### 3.2.6. Main Category 5: Navigation

Managers and skilled staff reported heterogeneous degrees of commitment to clients’ navigation in their facilities: to a low (5.3), medium (5.2), or high level (5.1). Facilities providing a high level of navigation support are characterized by using several different types of aids to facilitate navigation (e.g., a clearly designed homepage, signs, or color codes). Some of the facilities had at least one navigation system or structure (e.g., signs, contact staff) that served as an aid for navigating through the organization and, thus, provide a medium level of navigation (5.2). Particular importance is given to the presence of staff in those facilities which provide information about their facility, as well as offering individual tours and accompanying clients through their facilities. Some of the respondents also reported that navigation aids were scarce (5.3). However, structural improvements were planned to improve the navigation in those FPD in the future (5.1 and 5.3). Interviewees had different ideas about navigation tools in their facilities. In FPD in which navigation aids were available, interview partners reported, for example, that signs in easy language, pictograms, color codes, a clearly designed homepage, or flyers for clients and their relatives were present (5.4). For most interviewees it was not obvious that an inadequate navigation system can represent a barrier for clients or relatives and that a good guidance system represents an attribute of HLO. In addition, wishes and prospects for an improved guidance system were reported (5.5.).

#### 3.2.7. Main Category 6: Client Support in Health-Related Issues

Client support is provided in various health-related issues and is of central importance. Support ranged from the provision of medication to arranging and accompanying clients to medical appointments (6.1). Support often includes health services that are individually adapted to the needs of clients (6.2). The focus is on individualized offerings by skilled staff based on the degree of disability and the diagnosed needs of clients. The interviewees reported that the support design for clients is just as individual as the offerings itself. Thus, action is realized according to the situation and the individual client. To provide support in health-relevant issues, an extensive knowledge of and experience of the individual client is indispensable, since empowering the client entails providing only as much information as necessary. Various instruments and media (e.g., pictograms) are also used in supporting the clients on health-related topics (6.3). This enables clients to better understand their situation and express their needs or feelings to be better understood by skilled staff. 

#### 3.2.8. Main Category 7: Initiatives to Promote HL

All interview partners offered and strongly emphasized the importance of training for skilled staff. However, the topic of training varied greatly between FPD. Two areas of training were identified: firstly, training on client needs related to HL (7.1), for example, in the use of easy language; and, secondly, training for skilled staff related to the promotion of HL (7.2). In the first area, FPD offered a wide range of courses for clients. The second area addressed the training for skilled staff, which was comparatively less well developed and varied greatly among facilities. Training for staff included, for instance, further training on health-related subjects in general or the provision of health promotion for skilled staff. In addition, interviewees reported highly heterogeneous wishes for the expansion of training for skilled staff, including training in non-violent communication [48], easy language [47], and specialist information on dementia or healthy nutrition (7.3). The aim in FPD was, for example, to expand the range of services on the subject of HL and thus to also contribute to clients’ HL: *‘(...) if they do, it is more targeted at the health literacy of the skilled staff, but in the end, it also transfers directly to the unskilled employees, whether psychological or physical well-being’* (FPD 3, person A). In other FPD, the focus was on the expansion of the range of training, such as training in communication skills between skilled staff and clients. 

#### 3.2.9. Main Category 8: Health Promotion for Staff 

Different forms of health promoting initiatives for skilled staff were reported. However, some interviewees did not report any health promoting initiatives for skilled staff. Concerning services with a preventive and health-promoting character (8.1), the interviewees stated that they offer opportunities for skilled staff to use rehabilitation or fitness centers. Other facilities have company sports programs and offer lectures on specific clinical diseases or anthroposophical topics. One facility attached particular importance to the inclusive character of sports facilities where clients can train together with skilled staff, e.g., for running competitions. In connection with offerings for disease management and health care (8.2), interviews highlighted that, if needed, an in-house physician, company physician, or colleague serving as a first-aider was involved. In some of the facilities, a broader implementation of health services (8.3), their extension, or improved use in existing structures was desired. In this context, the potential for improving access to healthy food and stress prevention was mentioned. In some facilities, rigid rules and organizational inflexibility were seen as an obstacle to the implementation of health services for skilled staff. 

#### 3.2.10. Main Category 9: Health Services for Clients 

In comparison with the health-promoting initiatives for skilled staff, the initiatives for clients were found to be mostly behavioral-oriented and more extensive. All facilities provide exercise and sports facilities for clients, ranging from inclusive dance courses, swimming, and football, to active breaks and relaxation groups (9.1). Clients also had the opportunity to attend lectures and seminars on topics such as dementia, biographical work, or cooking courses. Some sports activities are not only organized internally together with skilled staff, but also externally in cooperation with local (sport) clubs. Although most of the facilities provide a balanced diet, it is difficult to implement healthy eating habits. The main difficulty in this context is that harmful nutritional behavior can take place outside the facilities’ radius of action. Nevertheless, FPD also reported the use of offerings or activities to promote a healthy diet (e.g., the free provision of water or fruits). Offerings aimed at disease management and health care (9.2) are also available for clients (e.g., physiotherapy, therapeutic eurhythmy, psychosocial counselling, assistance with treatment and prevention appointments, and support with their organization and preparation). These services are offered by company doctors, in-house medical services, or in-house therapy options. Some interviewees would like to see a wider range of relaxation or sports activities for their clients (9.3). These should be low-threshold, include internal offers, and take place regularly. Some of the facilities already have plans for restructuring and expansion. 

In sum, qualitative results showed that interview partners were partially aware of OHL, but they strongly focused on behavior-related health promotion. The implementation of HL in the mission statement or in the structures of the facilities was mostly indirect, as it had not yet been integrated into processes and procedures (e.g., in the form of guides). In particular, the area of navigation in FPD should be further expanded. Individualized health information has been developed in a participatory manner only to a low extent in the facilities for people with disabilities. In this context, individualized health information for clients was provided exclusively by skilled staff. This indicated that necessary information on health, among other topics, requires participatory development and skilled staff should be trained in health information, health communication, and OHL in general. 

## 4. Discussion

### 4.1. Summary of Quantitative and Qualitative Results 

The purpose of this study was (1) to adapt an instrument (i.e., HLHO-10) for measuring OHL to facilities for people with disabilities, (2) to assess the 10 attributes of HLHO quantitatively in those facilities, and (3) to qualitatively examine the definition and role of OHL from the perspective of managers and skilled staff with leading or health promotion responsibilities in Germany. 

Regarding the research question 1 on the adaption of the HLHO-10 instrument, study results revealed that the instrument of HLHO-10 can be easily adapted to other settings. The adapted HLHO-10-instrument that was used in this study was well-suited for facilities for people with disabilities. The slightly adapted questionnaire was appropriate for being used in organizations other than hospitals or health care organizations. In particular, the attributes of HLHO-10 referring to client’s involvement when developing health-related information or whether communication in facilities is targeted to client needs is very closely related to specific characteristics of settings where people with disabilities live and work.

Second, the findings of the quantitative study on the attributes of HLHO (research question 2) revealed that more than half of respondents in facilities for people with disabilities reported a below-average level of OHL (see Figure 3). Particularly with regard to involving clients in developing health information and the use of individualized health information, respondents reported a below-average level of OHL. Our findings suggest that improving OHL in facilities for people with disabilities is important, particularly in the area of strengthening the participation of people with disabilities in health-related issues.

Third, findings of the qualitative study (research question 3) highlighted that representatives of the facilities reported a very different and, in some cases, a complex understanding of OHL. An elementary understanding of OHL was found when interviewees referred, for instance, to the promotion of health-related behavior of clients and skilled staff. Interviewees with a more complex understanding perceived OHL as providing structures and means to promote individual health development of clients and their ability to act independently when necessary, as well as including staff training in health-related issues. 

The comparison between the quantitative and qualitative results showed that attributes of an HLO (HLHO-10) differed between main categories from the qualitative content analysis, indicating that some attributes of the original HLHO-10 instrument were less reported in the qualitative interviews. These differences between the quantitative and qualitative results are discussed for each main category from the qualitative study in conjunction with attributes of an HLO (HLHO-10) below. Due to the change of the main categories (see Section 2.2.4.), some attributes of an HLO (i.e., HLHO-item 5, 7–9 under 4.5. Health communication in facilities for people with disabilities) are discussed under one main category of the qualitative findings. Overall, qualitative and quantitative findings of this study highlighted that some attributes of OHL in facilities for people with disabilities were already developed to an average level and provided a good basis for the development of HLO. In particular, this included the range of support for health-relevant topics and the training of skilled staff, as well as individualized communication targeted to client needs. In addition, most interviewees were aware of the relevance and significance of both individual and organizational health promotion programs, which also provides important groundwork for establishing HL-promoting structures.

### 4.2. Discussion of Quantitative and Qualitative Results 

A discussion of findings in comparison to previous research is difficult, as there has been little research on OHL in facilities for people with disabilities in general. However, there is evidence from other studies on OHL which can be referred to as a point of departure for discussing the results of this study. 

*Implementation of OHL into the mission statement and as a task of leadership:* Implementing OHL in the mission statement or company objectives is of great importance for all stakeholders in an organization [27,30,49]. In particular, the commitment of the organization, and primarily at the management level, forms the basis for the establishment of OHL [27,33]. Even if, for example, facilities with an anthroposophical model did not refer to HL as such, the qualitative findings showed that a holistic understanding of a client as a health-literate person is already beneficial for HL. Results of the quantitative study supported this result as the mean value of the HLHO-item 1 on the leadership commitment to HL was above average. Previous studies repeatedly showed that a high HL of managers has a considerable effect on the health of employees [28,33]. In other studies, support provided by managers was crucial to implement OHL [25,33]. This is shown, for example, by a study using semi-structured interviews with 11 persons who were active in the management of health care organizations in the United States and successfully implemented HL changes, practices, and guides [25]. The participants in that study emphasized that the support of managers and administrative staff was indispensable for the successful implementation of OHL measures. It can therefore be assumed that barriers to a successful implementation of OHL (as described by Farmanova et al. [33]) depend mainly on the management level and there should be a fundamental openness to the topic of OHL. Further, it has been shown that organizations benefit when HL is an integral part of all organizational processes [23]. In general, it is advantageous for the implementation of HL when there is a person with necessary knowledge and skills who serves as an ‘HL champion’ [23,25]. An involvement of an (external) expert’s responsibilities for OHL can facilitate the introduction and acceptance of implementing OHL measures in organizations [23]. 

However, findings of our study further showed that awareness and implementation of OHL is systematized only to a small degree. According to the literature, the implementation of OHL should focus on formal instead of informal initiatives [18]. This is a result from a study with *n* = 40 medical staff (including nurses, doctors, chemists, and office workers) in Italy on the awareness and implementation of HL in their health care organizations [18]. Formal initiatives are planned and supported by the organizations themselves. Informal initiatives, on the other hand, are measures that are carried out spontaneously by skilled staff without any specific organizational incentive. Although formal procedures are perceived to be more effective, informal initiatives are carried out more frequently. The formal commitment of health care organizations to the promotion of OHL is therefore considered essential [18,22,33,37]. In addition, resources play an important role when promoting OHL: participants working in (health care) organizations with limited capacities (e.g., financial resources, leadership commitment, personal resources due to high turnover) were less enthusiastic and energetic in addressing changes to HL. Lack of funding is seen as the biggest obstacle to organizational change and its establishment, as shown in other studies [22,25,33].

*Implementation of OHL in the QM:* In the area of QM, a clear need was identified in the qualitative data, but not the quantitative data, as the mean value of the HLHO-2 item was above average. One reason for this could be that respondents in the quantitative study considered health promotion measures as part of QM per se. They did not differentiate between health promotion activities in facilities and the concept of HL. In order to increase health knowledge and health behavior of skilled staff and clients, health-literate communication processes and structures as part of the QM are needed. In order to establish sustainable and high quality structures and processes, the individual quality dimensions (the structural quality of HL, its process quality and outcome quality) have to be measured, validated, and piloted [27]. When anchoring these processes in organizations, however, it has to be ensured that skilled staff are not burdened with additional work, as this in turn can lead to skilled staff becoming resigned to processes and efforts aimed at strengthening OHL [25]. 

*Participatory development of health information and individualized health information*: The mean values for the item “development of individualized health information” (HLHO-3) and the item “providing individualized health information” (HLHO-4) were below average in the quantitative online study compared to other dimensions of OHL. These findings are in line with the qualitative findings, as the development of health information (e.g., in the form of material and flyers) was reported as less relevant and had only been occasionally applied in facilities. The reason may be due to the circumstance that the management board puts more emphasis on adapting pre-existing health information to individual clients and occasions rather than focusing on collaboratively and participatorily produced material on health information. Overall, the adaption of health information to the target group specific needs is of central importance to the understanding of health information [50,51]. A meta-narrative literature review showed that people with disabilities have different “roles” in the initiation, design, and evaluation of accessible information [51], ranging from no participation [52] and being involved in the proof of comprehension [53,54], to client’s participation as a co-researcher [55,56]. Other studies emphasized that only strengthening functional HL in people with disabilities is an insufficient measure, as functional literacy is limited in people with intellectual disabilities, in particular [57,58]. In this context, strengthening the ability of clients to act independently with regard to their health is seen as central. Empowering clients to independently obtain the health information they need from other health care organizations enables them to have an active role in making decisions in health-related issues and obtaining help if necessary. However, due to the great variety of information sources, decision making regarding health-related concerns proved to be especially problematic among people with disabilities, as confirmed by previous studies [8,10,57]. In those studies, the search for health-related information on disease management was less relevant for people with disabilities [8,10,57]. Thus, it is important that facilities for people with disabilities develop health information participatorily that is target-group specific to provide relevant health information, to support clients’ ability to understand information correctly, and to enable them to make health-related decisions as independently as possible. 

*Health communication with different media in facilities for people with disabilities:* Regarding the results on health communication in facilities for people with disabilities, the quantitative results showed an above-average mean-value of this item (HLHO-5). This is consistent with the qualitative results showing that skilled staff are an important source of information for clients. A client’s ability to understand health-related information is considered to be particularly important. Since there are no adequate communication standards for people with disabilities, facilities focused on communication adapted individually to client needs, including knowledge of easy language and educational skills. This might explain why respondents revealed to provide client information in different media only to a low degree (HLHO-7). Results of the qualitative and quantitative (HLHO-8 and HLHO-9) study highlighted that the client’s understanding was always emphasized by skilled staff, especially in critical situations and financial decisions. The area of health communication, in particular, represented an important, but also challenging, area in the promotion of HL [28]. Some studies highlighted the importance of individually tailored health information and communication between clients and specialists [10,51,57]. Chinn [19] notes that this area is of particular relevance, because the better health communication is structured, the fewer the rate of false diagnoses, problems in disease treatment, or occurrence of secondary diseases [4]. By providing better health communication, the coordination and organization of doctor and hospital visits for the organization will be simplified [4].

*Navigation throughout and in facilities for people with disabilities:* The quantitative and qualitative results regarding issues of navigation and “efforts made to help clients easily find their way” (HLHO-6) were very diverse. Quantitative findings showed an above-average and the highest mean value in navigation compared to other HLHO-10-items. In contrast, results of the qualitative interviews showed that there was a clear need for improving navigation systems, which was not recognized by all interviewees. Facilities for people with disabilities seemed to view themselves as a closed system and therefore consider navigation within facilities to be more relevant. However, concepts of OHL also emphasize the access to organizations as a particularly relevant attribute of OHL [59]. Information material on orientation, and maps to and through organizations (e.g., published on websites or via flyers), are also of particular importance for future stakeholders, such as clients and their relatives.

*Health promotion offers and training for skilled staff and clients*: The quantitative and qualitative results revealed that there was still a need for improvement in the area of training for skilled staff on HL (HLHO-10). Qualitative results showed that health promotion offerings and training for skilled staff and clients was also emphasized. These results indicate that facilities for people with disabilities are meeting the demand for distributed HL [60] in relation to people with disabilities [57]. By doing so, health promotion offerings, as well as staff training in the area of health and communication, are likely to strengthen health awareness and health knowledge in the entire setting, thus contributing to an increase in HL for all participants, both for skilled staff and for clients.

## 5. Limitations

This study is the first study, to our knowledge, that provides quantitative and qualitative information on OHL in order to assess the understanding and characteristics of OHL in facilities for people with disabilities in Germany. Even though the initial aim of the quantitative online study was to asses OHL in facilities for people with disabilities in Germany, the response rate was very low, only 4.78%. Other surveys—especially online surveys—report higher response rates of 10% to 80% [61,62]. There are several possible reasons for the low response rate in this study, as participation in online surveys is influenced by various factors, such as the intended use of the survey by respondents, delivery mode and access, survey length, or interest in survey topic [63]. However, findings of the quantitative study may constitute suitable groundwork for future studies. Although the quantitative study did not apply a representative sampling and was based on a convenience sampling procedure, results cannot be generalized to all facilities for people with disabilities in Germany. However, results can contribute to further development of quantitative instruments for assessing characteristics of OHL in facilities other than health care organizations. 

It should also be noted that the qualitative study was conducted at the regional level, in the German federal state of Hesse. In order to generate a typology of different roles of OHL features, an increase in the number of interviews is recommended in future studies. By doing so, it would be possible to quantify codes in the interview material, which was not part of the coding process of this study. A typology could then contribute to develop target-group-specific measures to strengthen the OHL in facilities for people with disabilities.

A further limitation results from the complex concept of OHL used in the qualitative study. It should be questioned whether the definition of the term OHL that was presented to interview partners at the beginning of the interviews was sufficient to form an adequate understanding of OHL. During the entire research process, attention was paid to the quality criteria of qualitative research. Subsequent research is required to extend the sample of facilities for people with disabilities to other German federal states or other countries.

## 6. Implications to Strengthen OHL in Facilities for People with Disabilities

As facilities for people with disabilities work with a vulnerable population group, measures to promote HL should concentrate not only on employees and skilled staff but also on their clients with disabilities. In the following section implications are given in order to implement and strengthen characteristics of OHL in facilities for people with disabilities according to the results of our study. In general, it is recommended to use both individual and organization-level measures not only to implement an intervention to improve OHL, but also to use different methods in combining complementary measures [59,64,65]. In order to establish OHL in facilities, it is required to form an OHL team consisting of different stakeholders of the organization. This OHL team should assess different features of OHL [29]. Instruments that can help to develop an HLO are, for example, the 18 attributes developed by Hernandez et al. [66]. These attributes include, for instance, needs-based resource distribution, patient training, barrier-free health information technologies, the prioritization of drug safety and communication, the support of patients/clients and their relatives to find their way around the health system, and the creation of an open and questioning culture. In addition, there are various instruments available for physicians and employees in a clinical work setting that may help make the organization health literate. These range from self-assessments, online training and assessment measures to staff surveys [22]. 

Results of this study revealed differences in the mission statement and understanding of the meaning of OHL between interviewees. A first step to establish and improve OHL in facilities should be to prioritize HL and OHL in the leadership and mission statement of facilities. Further, facilities are encouraged to address the awareness of OHL among clients and staff. This could be achieved, for instance, by events on HL, through health education in clients, role plays regarding different health topics, or training for employees in facilities [26]. 

Regarding individualized health information, it is necessary to develop health information in a participatory manner in close collaboration with clients in order to overcome the current state recognized in most previous studies, namely, that health information is not being developed in a participatory way [51]. Another possibility to consider clients as important stakeholders is in getting feedback on material or organizational features on OHL by clients and their relatives, for example, by installing a feedback box [29]. Further, previous studies on promoting OHL highlighted that health-related information should be given to clients both in verbal and written form. In particular, it is recommended to work with visualizations such as pictograms to help people with disabilities, particularly those with limited reading skills, in understanding health relevant information [5,59,67]. For instance, one study revealed that almost all patients, especially those with limited HL, benefited from an illustrated medication schedule [67]. 

With regard to the dimension of health communication it is important to improve skills of (health) communication among clients and staff. Communication techniques such as Ask-Me-3 [68] or the Teach-Back Method [29] have proven to be effective in individualizing health communication in health care organizations. For instance, by using the Ask-Me-3 method [68], clients learn to ask three questions to better understand their state of health: “What is my main problem?”, “What do I need to do?”, and “Why is it important for me to do this?”. They can ask these questions themselves or address them to the health professionals in their facility or at the doctor’s consultations. Clients should be encouraged to ask these questions and to participate actively in discussions about their health. This will further enable them to take actions and responsibility for their own health. The Teach-Back Method, for example, requires the clients to repeat in their own words what has been said in order to check whether they have correctly understood the health-related information. Those methods could also be included in degrees in human medicine and nursing education so that future health staff will be able to break down barriers in health communication from the very beginning, so that health literacy is more likely to be strengthened in the long-run [14,34,60]. Further, it is also recommendable to encourage clients to ask questions about their health, treatment, etc. Regarding (medical) staff, it is also crucial to prepare and train doctors and medical skilled staff. Hence clients should be enabled to practice communication skills that are likely to reduce comprehension problems during appointments. Clients should be enabled and empowered to ask for explanations in easy language in order to better understand and apply medical advice. By doing so, this might enable clients to be more independent and health-conscious as they are their own health experts [4,19]. A further implication addresses the need for communication training for skilled staff and employees that not only includes developing communication skills, but also includes reflection on human rights and one’s own perspective on people with special needs. Lastly, it is also important to create health-friendly and safe settings for clients that motivate them to make health enquiries in order to promote health-literate communication [4,5,19].

With regard to the attribute of navigation as part of OHL, this feature can be improved by guaranteeing access to and navigation in and between buildings through, for instance, clear sign systems with easily understandable symbols and provided in easy language [22]. Groene and Rudd [59] used the example of hospitals in Spain to show that there are major differences between hospitals in this respect. For instance, hospitals used different visual materials (e.g., signs, symbols, pictograms, 3D models, photographs of skilled staff members, and patients in the corridors or at doors). Further, the complexity and degree of scientific terminology in written information and the uniformity of terms used by staff for the same issues was heterogenous [59]. Another way to improve navigation is to train skilled staff to be able to respond competently to questions about directions [5]. In designing a navigation system that meets the criteria of an HLO (see e.g., Brach et al. [22]), a number of studies implement the “First Impression Toolkit”, using the “First Impression and Walking Interviews” [69] on site and in experiencing the telecommunications services (i.e., website, telephone) of the organization [70,71]. Members or external persons of the organization have to take a walk through the buildings and on the premises, while completing a number of tasks that enable them to perceive their organization with, as far as possible, the eyes of a “stranger”. They thereby are likely to detect barriers and missing information (both in access to and by walking through the organization).

In addition to the implications for facilities for people with disabilities, the responsibility of (political) decision-makers for strengthening HL should also be mentioned [5]. The first priority at the political level should focus on creating awareness of HL. Since HL and education are closely related, the promotion of HL in the educational sector should be strengthened, especially for children and adolescents with disabilities, but also in adult education [5,72]. 

Emphasis should be given to the development of specific toolboxes for strengthening characteristics of OHL, such as by the research project “EwiKo” (“Entwicklung von Gesundheitskompetenz in Einrichtungen des Gesundheitswesens”/“Developing Health Literacy in Health Care Organizations”; 2020–2023). This project aims at developing and establishing structures for a sustainable improvement of organizational health literacy in hospitals, care organizations for elderly people, and facilities for people with disabilities in the German federal states of Saxony and Thuringia. The focus of this projects is on the participatory development, implementation, and evaluation of interventions to promote individual and organizational health literacy. Lastly, a digital transfer concept for the dissemination and implementation of the project results by multipliers will also be created in order to improve individual and organizational health literacy among staff and clients in those settings in the long-run.

## 7. Conclusions 

The main aim of this study was to examine characteristics of OHL in facilities for people with disabilities in Germany, using a mixed-methods design. The first aim of this study was to adapt the instrument of HLHO-10 to the context of facilities for people with disabilities. As this original instrument provides 10 short single questions on the assessment and level of OHL, this instrument was modified only slightly by changing some terms after having applied two pre-tests with representatives of two different facilities for people with disabilities. Thus, the HLHO-10 can easily be adapted to settings other than hospitals or health care organizations. 

With regard to the second aim, the quantitative online study revealed that characteristics of OHL were, on average, at a medium level. With regard to single characteristics of OHL, results of the quantitative study further showed that respondents reported a below-average level of OHL in individualized information for clients and participatory development of health information, whereas navigation scored at an above-average level. 

As part of the third research aim, the qualitative study revealed that the definition and role of OHL in facilities for people with disabilities, as well as the complexity and understanding of OHL, differed between interviewees. A basic understanding of OHL was found in all facilities. However, the awareness that OHL is likely to be promoted through structural adjustments, by means of health promotion, strengthening the independence and personal responsibility of clients in health-related issues, and staff training, was only found in a few interviews. Overall, this study highlights that the involvement and participation of the target group (i.e., people with disabilities) and their relatives, as well as skilled staff, is crucial for the planning, implementation, and analysis of HL-promoting measures, particularly in facilities for people with disabilities. 

## Figures and Tables

**Table 1:** Sample description of the quantitative and qualitative survey.

	Qualitative Survey		Quantitative Survey	
	Frequency in		Frequency in	
	*n*	%	*n*	%
**Sex of Respondents**				
Male	5	50.0	45	34.6
Female	5	50.0	85	65.4
Total	*n* = 10		*n* = 130	
**Age of Respondents**				
18–30 years	2	20.0	3	2.3
31–40 years	1	10.0	30	23.1
41–50 years	3	30.0	43	33.1
51–60 years	3	30.0	42	32.3
61 years and older	1	10.0	12	9.2
**Position in the Organization**				
Executive	1	10.0	5	3.8
Manager	6	60.0	108	83.1
Skilled staff	2	20.0	6	4.6
Other	1	10.0	11	8.5
**Financing**				
State (municipal or federal)	3	37.5	16	12.3
Non-state/church	2	25.0	90	69.2
Private or other	3	37.5	24	18.5
**Type of Organization**				
Housing for people with disabilities	3	37.5	96	73.8
Sheltered workshops	3	37.5	34	26.2
Combination of housing and workshop	2	25.0	-	-
**Number of Skilled Staff**				
Fewer than 50	3	37.5	77	59.2
More than 50	5	62.5	53	40.8
**Profit or Non-Profit Facility**				
Non-profit	7	87.5	114	87.7
For profit	1	12.5	16	12.3

**Table 2:** Descriptive statistics of the organizational health literacy (OHL) attributes (*N* = 130).

HLHO-10 Items	M	SD	Min.–Max.
1. Is the leadership explicitly committed to HL (e.g., mission statement, HR planning)?	5.2	1.4	1–7
2. Is HL in your organization integrated into quality management measures?	5.0	1.5	1–7
3. Are clients involved in developing health information in your organization	3.9	1,7	1–7
4. Is individualized health information provided by your organization (e.g., other languages, large print, braille)?	3.9	1.9	1–7
5. Are there communication standards in your organization that ensure patients actually understand the information they need (e.g., translators, time to think before answering, asking for questions)?	5.0	1.6	1–7
6. Does your organization make an effort to ensure clients can easily find their way (e.g., signposts, health information staff)?	5.4	1.5	2–7
7. Is information in your organization available for different client groups in different media (e.g., 3D models, DVDs, illustrated stories)?	4.1	1.9	1–7
8. Does your organization ensure that especially in critical situations your clients actually understand everything (e.g., intake of medication, therapeutic measures, patient consent)?	5.2	1.5	1–7
9. Does your organization assist clients in determining possible costs (e.g., additional payments for medical or other health-related treatments)?	5.3	1.8	1–7
10. Do employees and skilled staff in your organization receive training in HL?	4.8	1.6	1–7
Average mean of HLHO-10: overall degree of organizational health literacy	4.8	1.1	2–7

M: mean value, SD = standard deviation.

## Appendix A

**Table A1:** Detailed codebook for qualitative content analysis with anchor examples (according to Kowalski et al. 2015 and based on HLHO-10 questionnaire).

Category	Subcategories	Definition	Anchor Examples
1. Mission statement		The facility’s leadership is explicitly committed to HL (e.g., mission statement, HR planning).	
	1.1. High level of HL in mission statement	Strongly anchored in mission statement or concept, part of processes, defined objectives, or liabilities	‘It’s an integral part of our concept.’ (FPD 4, person B)
	1.2. Medium level of HL in mission statement	HL is anchored to an extent in the mission statement; not as a binding objective or element in the concept; plays a relatively insignificant role or has a different designation	‘But in writing I have to say that it’s only a small part.’ (FPD 1)
	1.3. HL is not part of the mission statement	HL is not structurally embedded; the concept does not include any special requirements or rules regarding health	‘(…) There are no completely integrated (…) no real processes anywhere that are a real part of the concept. That just isn’t happening.’ (FPD 3, person A)
	1.4. Implicit anchoring and implementation of HL in mission statement	HL without conceptual requirements or binding objectives; health-relevant offerings or measures are voluntary and out of the interest of the skilled staff; part of a philosophical mission statement based on anthroposophical principles and holistic spiritual background	‘There are many everyday processes that I would say might unconsciously lead to this way of health behavior.’ (FPD 3, person B)
2. Quality management		Is HL part of quality management in your facility?	
	2.1. Quality management in general	QM in crisis situations: ensures understanding of the situation, routine or standard processes; QM in working conditions, work safety; measures to improve and monitor working conditions	‘We have (…) trained first-aiders (…) and we have an internal direct number. So by a key combination on the phone (…) you start an internal emergency call when fast action is needed and first of all the first-aiders are on site and then it actually sets off a relatively fast chain reaction.’ (FPD 3 person B)
	2.2. HL in Quality management	HL in quality management for working and living together; relevance of HL in the facility regarding possible conflict in operations or care; measures to prevent or availability of contact points; quality management measures that are aimed at ensuring medical care or supporting HL during medical interventions	‘We have two colleagues who are trained in de-escalation techniques and how already told, they share their knowledge with the skilled staff. All employees are required to take part in this training.’ (FPD 3, person B)
3. Clients involvement		Are clients involved in developing health information in your facility?	
	3.1. Participatory	Clients are involved in developing health information.	-
	3.2. Non-participatory	Clients are not involved in developing health information.	‘Up to now, this has mainly been due to the intuition of the employees. So, in principle, the employees always have a feeling for how my clients tick, what their interests are.’ (FPD 8)
4. Communication standards		Are there communication standards that ensure that clients understood critical information (e.g., translators, pause to allow time to think before responding, encourage questions)?	
	4.1. Implementation	Design of the communication between skilled staff and clients	‘So, that always has to be individual and it depends on the person.’ (FPD 3, person B)
	4.2. Influences	Factors influencing the communicative situation, such as critical situations or individual clients’ limitations	‘So, we always have appointments with the clients. Time appointments. (…) And during this time (…) There are always situations that make you see that you should really stop for a moment and really take more time for a topic. But I really don’t have the time because I already have to go somewhere else. It’s also a question of relationships whether you can put something off to another day. (…) The better your relationship is to a client, the better the possibility to communicate in this area. It’s very much about trust. (FPD 5)
	4.3. Basics	Use of communication technologies and media	‘Violence free communication and easy language.’ (FPD 5); ‘Use picture.’ (FPD 2)
5. Navigation		Is an effort made in your facility to help clients easily find their way (e.g., signs, information staff)?	
	5.1. High level of navigation aids	A number of different aids to facilitate navigation are used, e.g., signs in plain language, pictograms, color codes, clearly designed homepage, flyers, information staff	‘We have a homepage with an app for your phone. So that you can use it on your phone. We have an area with easy language. We have signs in the building. We have prepared all light switches that they can be used by people in a wheelchair. Barrier-free access to all rooms. In each work area, we have a flyer which we provide for initial interviews (…) Then there is a general flyer. Then we have regular open house days and celebrations for our skilled staff and clients.’ (FPD 5)
	5.2 Medium level of navigation aids	There is at least one feature/tool that serves as a guide; for example, signs in plain language, pictograms, color codes, clearly designed homepage, flyer or information staff	‘And I think we have a very strong presence of staff offering guidance. (…) Guidance is, I think, relatively easy. (…) We don’t even have decent signs. Every external remembers this. But every person in care will be able to tell you where the wool workshop is. There is much (…) always this presence from people, someone is always there.’ (FPD 2)
	5.3 No navigation aids	There are no aids in the facility that explicitly serve as guidance	‘I would say, at the moment there isn’t a guidance system and it’s not clear at all, so in terms of the homepage and all.’ (FPD 1)
	5.4 Examples of navigation aids	Structures or systems that serve as guidance in the facility	‘Homepage’; ‘plain language app’; ‘flyers for each area.’; ‘Signs.’ (FPD 5)
	5.5 Wishes and prospects for an improved guidance system	Improvements to the system are planned or expressed as desirable	‘We are working on what offerings we have in this area, what offers our homepage for navigation already, what’s missing, what we need, what relatives need, what our clients need, what outsiders need.’ (FPD 8)
6. Client support in health-related issues		In your facility, do you offer support in determining costs such as additional payments for medications or other treatments relative to health?	
	6.1 Offerings/services	Offerings that are supported, e.g., doctor appointments, health offerings	‘As a rule, we arrange an appointment with the doctor together with the clients. We call there together or the person calls and I help out depending on what the client can do. But the initiative has to be there. I can’t force anyone to go anywhere.’ (FPD 5)
	6.2 Type/kind of support	Design of the support mechanisms	‘That’s very diverse. It’s as individual as the people with us, as individual as the solutions. There are people who can hardly tell us about their illness, their pain, or other things. You have to observe very carefully to even see if they aren’t feeling well. And there are others that complain the whole day long about pain they don’t even have. You have to handle things on an individual basis and respond its.’ (FPD 7)
	6.3 Media use for health-related topics	Auditory (audio media), visual (print media), audio-visual (media with images or videos and sound), multimodal (media combining language and images)	Language, audio material (FPD 8); ‘There’s a pictogram for every condition: stomach aches, fever, everything. We have a little box in the rehabilitation workshop for those clients who are able to show us what’s wrong with them.’ (FPD 3, person B)
7. Initiatives to promote HL		Are skilled staff in your facility trained in HL?	
	7.1 Client-related training	Offerings training and further education related to special diseases or activities affecting clients; opportunities and resources for training in HL communication	‘We have different departments such as care sector, assistant area. Assistants are trained, they work individualized how the clients prefer. And we, in assisted living, where I work, some clients I care for have a special condition like diabetes or trouble with their heart. I have a client who has multiple such as problems with her kidneys, and I went to special training courses.’ (FPD 5)
	7.2 Staff-related training	Opportunities for skilled staff to inform themselves about HL, support for personally managing occupational health risks or developing a healthier lifestyle; measures, rules, or regulations with the aim of supporting skilled staff health	‘Yes, well, I think, of course, self-management is a good thing, to have a good perception of oneself, that is, good provision for oneself, and team discussions and development discussions with skilled staff is a bit of a thing we do.’ (FPD 2)
	7.3 Wishes and ideas about training	Concepts or ideas about an ideal HLO regarding skilled staff training; wishes for the establishment or expansion of training, or potential for development	‘If it would be a concert of desire, I think it would be important for every skilled staff to have more training in communication to learn how I can best reach my clients.’ (FPD 8)
	7.4 Planning and implementation	Modalities of training: type of training opportunities for skilled staff, optional or mandatory, frequency, regularity, external or internal training, dissemination in team, type of availability, implementation in HR development plans, origins and development of offerings	‘We have an internal training network and if something is in the program [brochure] then you can apply to take part in it if you want to.’ (FPD 3, person B)
8. Health promoting for staff		All offerings to improve health in skilled staff	
	8.1 Prevention and health promotion	Offerings to prevent, delay, or reduce the risk of health impairment as well as means for the prevention or early detection of disease; offerings to strengthen health and knowledge about improving health, and also to increase health-preserving factors; salutogenetic approach	‘The sheltered workshop offers a sport activity free of charge at the gym of your choice in your preferred region. You can just join it, which is already a very big offer, of course. Employers expect there will be less downtime because of back problems or whatever happens when you don’t get enough exercise. That’s really something.’ (FPD 4, person A)
	8.2 Disease management and health care	Offerings to support coping with the stress brought about by disease; psychological or physical support in coping with a disease and its consequences; support for offerings in health organizations, products, and services available within the health system and whose use involves cooperation with health insurance companies or other types of support	‘B2: And I think that’s a really great thing, and then there’s also such an offer for the non-disabled employees that you can turn to your colleagues even after traumatizing experiences at work. (…) B1: [We have| Colleagues as first-aiders (…)’ (FPD 3, person B1 and B2)
	8.3 Wishes and ideas	Specific wishes about new, more, or other health offerings; ideas about what offerings should be provided; need for changes or development; actual plans to restructure offerings; expanding or providing initial offerings for skilled staff	‘We should see if we could do and embed things like this without consulting with the board. To simply bring things into life more freely and faster and to implement them as well. Yes.’ (FPD 1)
9. Health services for clients		All offerings to improve health for clients	
	9.1 Prevention and health promotion	Offerings to prevent, delay, or reduce the risk of health impairment as well as means for the prevention or early detection of disease; offerings to strengthen health and knowledge about improving health, and also to increase health-promoting (environmental) factors; salutogenetic approach	‘Nevertheless, in sheltered workshops there are health promoting offerings like exercise, eating the right food we’re looking at here.’ (FPD 7)
	9.2 Health promotion and health care	Offerings to support coping with the stress brought about by disease; psychological or physical support in coping with disease and its consequences; support for offerings in health organizations, products, and services available within the health system and whose use involves cooperation with health insurance companies or other types of support	‘If an individual (client) needs, for example, cancer treatment, then of course we look into how they can be dealt with individually, what has to happen, what else can be done, whether it’s possible to use external offerings. To go with them. Are there doctors they will come to us if the client can’t leave our premises?’ (FPD 8)
	9.3 Wishes and ideas	Specific wishes about new, more, or other health offerings; ideas about what offerings should be provided; need for changes or development; actual plans to restructure offerings; expanding or providing initial offerings for clients	‘(…) We have a recreation room. That’s where we’ll have this relaxation group. (…) We also want to offer a day group, expand it significantly and offer a larger spectrum. Where things like (…) health literacy, sport offers, nutrition offers play a more significant role. So, those are all ideas that are being developed. (…) There are already scheduled days, there are already staff being hired, but it’s all starting first in the summer.’ (FPD 1)

FPD = Facilities for people with disabilities.
